# Use of population-based cancer registry data to evaluate organized breast cancer screening programmes in Europe by mode of detection: a scoping review

**DOI:** 10.1093/eurpub/ckag090

**Published:** 2026-06-17

**Authors:** Wendy Kam, Elodie Guillaume, Quentin Rollet, Dafina Petrova, Nicolás Francisco Fernández-Martínez, Koen van Herck, Marià Carulla, Claudia Pla, Francina Saladié, Cristina Miracle, Vesna Zadnik, Sonja Tomšič, Anne Cowppli-Bony, Guy Launoy, Claudine Backes

**Affiliations:** Public Health, U1086 ‘ANTICIPE’ INSERM—University of Caen Normandie, Bâtiment Biologie Recherche, CHU de Caen, Caen, France; Public Health, U1086 ‘ANTICIPE’ INSERM—University of Caen Normandie, Bâtiment Biologie Recherche, CHU de Caen, Caen, France; Public Health, U1086 ‘ANTICIPE’ INSERM—University of Caen Normandie, Bâtiment Biologie Recherche, CHU de Caen, Caen, France; Department of Precision Health (DoPH), Cancer Epidemiology and Prevention Group (EPICAN), Luxembourg Institute of Health (LIH), Strassen, Luxembourg; Registre National du Cancer du Luxembourg, RNC, Strassen, Luxembourg; Cancer Registry of Granada, Escuela Andaluza de Salud Pública, Granada, Spain; Escuela Andaluza de Salud Pública, Instituto de Investigación Biosanitaria ibs, Granada, Spain; Instituto de Investigación Biosanitaria, University of Granada, Granada, Spain; University Hospital Virgen de las Nieves, Department of Preventive Medicine and Public Health, Granada, Spain; Escuela Andaluza de Salud Pública, Instituto de Investigación Biosanitaria ibs, Granada, Spain; Belgian Cancer Registry, Universiteit Antwerpen, Brussels, Belgium; Cancer Epidemiology and Prevention Service, Hospital Universitari Sant Joan de Reus, IISPV, Reus, Spain; Cancer Epidemiology and Prevention Service, Hospital Universitari Sant Joan de Reus, IISPV, Reus, Spain; Cancer Epidemiology and Prevention Service, Hospital Universitari Sant Joan de Reus, IISPV, Reus, Spain; Cancer Epidemiology and Prevention Service, Hospital Universitari Sant Joan de Reus, IISPV, Reus, Spain; Slovenian Cancer Registry, Institute of Oncology, Ljubljana, Slovenia; Slovenian Cancer Registry, Institute of Oncology, Ljubljana, Slovenia; Belgian Cancer Registry, Universiteit Antwerpen, Brussels, Belgium; Public Health, U1086 ‘ANTICIPE’ INSERM—University of Caen Normandie, Bâtiment Biologie Recherche, CHU de Caen, Caen, France; Department of Precision Health (DoPH), Cancer Epidemiology and Prevention Group (EPICAN), Luxembourg Institute of Health (LIH), Strassen, Luxembourg; Registre National du Cancer du Luxembourg, RNC, Strassen, Luxembourg

## Abstract

Organized breast cancer screening programmes are a cornerstone of cancer control policy across Europe. Population-based cancer registries (PBCRs) play a central role in monitoring screening performance, outcomes, and programme quality. However, the extent and methodological approaches of registry-based evaluations across Europe have not been comprehensively synthesized. Following PRISMA-ScR guidelines, we conducted a scoping review of peer-reviewed studies evaluating organized breast cancer screening programmes in Europe using PBCR data. Studies were identified through systematic database searches and screened using predefined eligibility criteria. Data were extracted on study design, definitions of detection mode, outcome measures, and approaches to bias adjustment. The Reach, Effectiveness, Adoption, Implementation, and Maintenance (RE-AIM) framework guided the methodological analysis. Twenty-six studies were included, mainly from Western and Northern Europe, with most published before 2010. Screen-detected cancers consistently showed lower mortality, better survival, and more favourable tumour characteristics than interval or non-screened cancers. However, reporting of screening indicators was inconsistent. Definitions of detection modes, particularly interval cancers, and approaches to bias adjustment varied widely, limiting comparability. Few studies applied comprehensive bias correction or clearly reported registry–screening data linkage. Registry-based evaluations provide valuable evidence on the impact of breast cancer screening programmes, **Howeve**r, the evidence is limited by methodological heterogeneity and limited analytical standardization, reducing its comparability and policy relevance. Strengthening standardized definitions, improving transparency in analytical approaches, and better integrating programme monitoring with research could enhance the public health value of registry-based screening evaluation.

## Introduction

Breast cancer is the most frequently diagnosed cancer in women worldwide and remains a leading cause of cancer mortality [1, 2]. Population-based organized breast cancer screening programmes have been implemented across many European countries with the aim of reducing mortality through early detection [3]. Evaluating the effectiveness and quality of these programs is essential to ensure that screening achieves its intended public health benefits while minimizing potential harms [4, 5].

Population-based studies using cancer registry data are well suited to evaluate screening effects in the populations they cover, as registries provide the only exhaustive source of cancer incidence data. When linked with screening programme data, population-based cancer registries (PBCR) offer opportunities to assess screening outcomes in real-world conditions [6, 7]. PBCRs also support classification of breast cancer cases by detection mode: screen-detected, interval, and non-screened (mostly symptom-detected), each associated with distinct prognostic patterns [5].

However, the use of PBCRs for screening evaluation remains inconsistent across studies and settings, with substantial variability in how data are linked, analysed, and interpreted. Few reviews have systematically mapped how PBCR data have been used to assess breast cancer screening programmes across Europe. The recent launch of the European Union’s CancerWatch joint action highlights the importance of data linkages to strengthen the quality, comparability, and usefulness of PBCRs at the EU level [8].

This scoping review therefore aims to examine the scope, methodological approaches, and outcomes of studies that used registry data to evaluate screening programs by comparing cancers according to their mode of detection. In addition, we synthesize the epidemiological outcomes reported across studies, including mortality, survival, tumour characteristics, and screening performance indicators.

## Methods

A scoping review was conducted following the Preferred Reporting Items for Systematic Reviews and Meta-Analyses extension for Scoping Reviews guidelines [9]. The protocol for this study is available in Material S1.

### Search algorithm

Medline via PubMed and Embase were searched using algorithms defined by two researchers and one documentation officer:

PubMed: ((‘cancer registry’[TW] OR ‘cancer registries’[TW]) OR (Registries[MH] AND Neoplasms[MH]) OR (cancer [TW] OR cancers [TW])) AND (Breast Neoplasms [MH] OR ‘Breast Cancer’[TW]) AND (Mass Screening [MH] OR ‘Screening Program*’ [TW] OR ‘Cancer screening*’ [TW]).

Embase: (exp mass screening/or mass screening.ti, ab. or screening program$.ti, ab. or cancer screening$.ti, ab.) and (exp cancer registry/or cancer registr$.ti, ab.) and (exp breast tumour/or breast cancer.ti, ab) and limit 26 to ‘remove medline records’

On 3 March 2023, these queries returned 1020 articles from PubMed and 490 from Embase. Because the use of population-based cancer registry data is not always explicitly reported in article titles or abstracts, the search strategy combined both controlled vocabulary and free-text terms. Nevertheless, studies using registry data that did not report this information in searchable fields may not have been captured.

### Screening phase

Four researchers collaboratively defined a protocol outlining the inclusion and exclusion criteria (Material S2).

The screening phase was conducted in three steps using the Rayyan online tool [10].

Step 1. Eleven researchers independently screened the titles and abstracts of 40 randomly selected articles to test the relevance of the criteria and the reliability of decisions. Agreement reached 67.5%. Data were then unblinded, disagreements were discussed and resolved, and the criteria were refined, as shown in Material S2.

Step 2. Seven reviewer pairs were created, each randomly assigned 213 records for blinded title and abstract screening. Agreement reached 89%. After unblinding, each pair discussed their remaining disagreements, resulting in 98% agreement. All unresolved disagreements were passed to Step 3.

Step 3. A team of six researchers selected the final corpus through full-text screening and resolved remaining conflicts. Only an unblinded phase was used, and all conflicts were resolved, achieving 100% agreement.

After screening, the 183 remaining articles were categorized by evaluation approach:

1. Studies comparing cancers by detection mode (screen-detected, interval, non-screened),
2. Studies comparing cancers before and after the introduction of organized screening programmes,
3. Studies evaluating European screening performance indicators.

**Figure 1.**
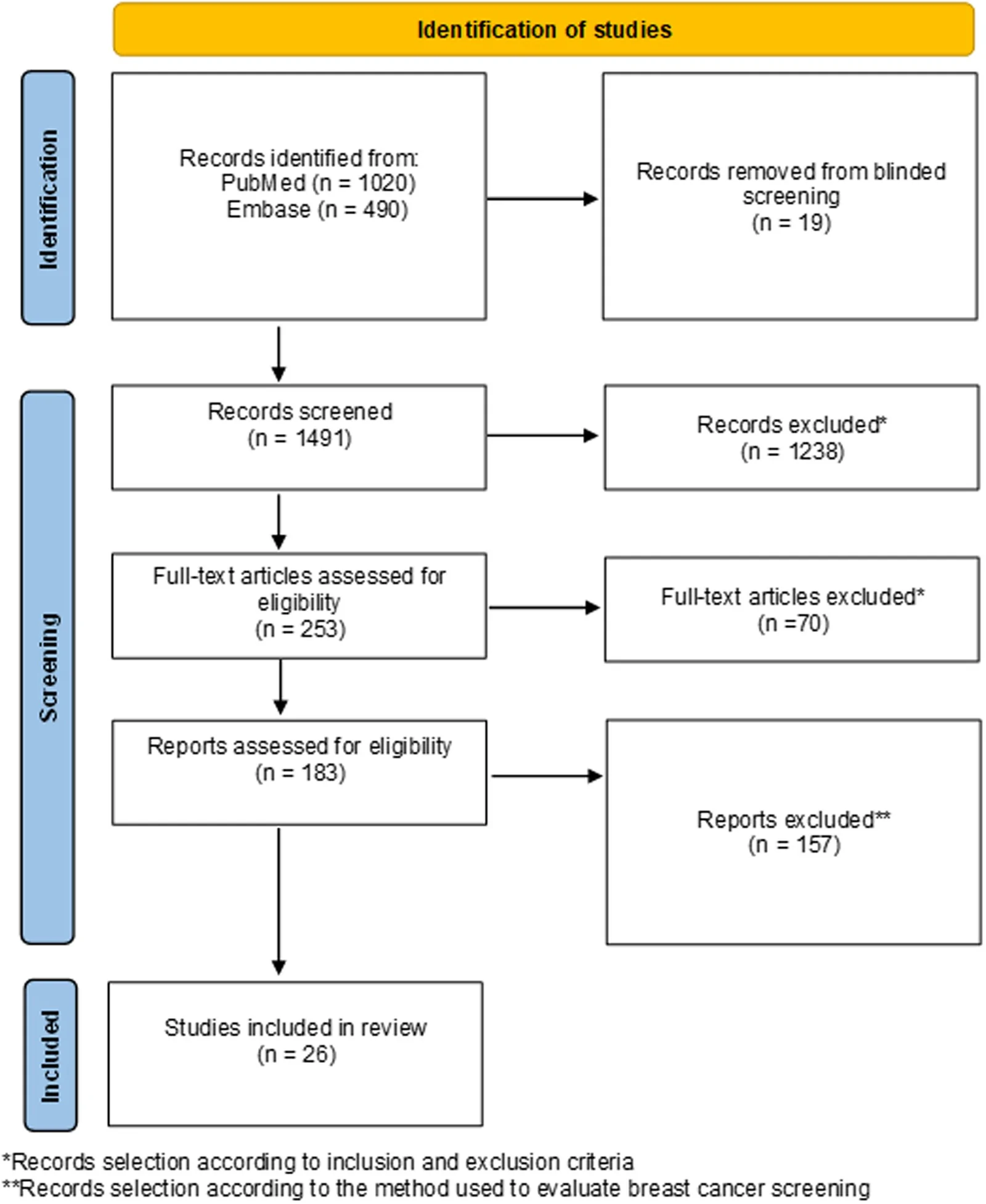
PRISMA flow chart of the selection process.

The present review focuses on studies comparing breast cancers by mode of detection. Applying this criterion resulted in the inclusion of 26 studies (Material S3). The remaining studies were retained for future analyses. This separation was necessary because the overall corpus was too heterogeneous to be synthesized within a single review (Fig. 1).

### Extraction phase

The extraction phase was conducted in three steps.

Step 1. A first version of the extraction grid was drafted by one researcher and circulated to the others for initial comments.

Step 2. A team of six researchers refined the grid based on these comments, and a test was performed on 10 included articles to ensure the relevance of the items and the consistency of extraction, resulting in the final grid (Material S4).

Step 3. Data were extracted in pairs, with one researcher responsible for extraction and the other for validation.

The extraction grid captured variables directly related to the research questions. It included study characteristics (authors, year, country, aim), screening data, registry data features, linkage approaches, and outcomes such as tumour characteristics, survival, mortality, and screening quality indicators. It also captured reported bias adjustments (e.g. for lead-time or socioeconomic status). This structure allowed systematic comparison across the heterogeneous literature.

#### Analysis

To guide the analysis, the RE-AIM framework (Reach, Effectiveness, Adoption, Implementation, Maintenance), a public health model supporting comprehensive evaluation of health interventions in real-world settings, was applied [11].

Each dimension of the RE-AIM framework was mapped to specific study aspects:

- Reach: definition of screening exposure and representativeness of screened populations;
- Effectiveness: captured clinical outcomes such as mortality, stage at diagnosis, and prognostic indicators;
- Adoption: settings and institutions conducting the studies, including registry types;
- Implementation: procedures used to classify detection modes and standardization of methods;
- Maintenance: sustainability of registry-based screening evaluations over time.

Detailed information and measures of the RE-AIM framework adapted to the included studies are reported in Table 1.

**Table 1.** Characteristics of the studies included in this review, based on the RE-AIM framework

Study ID/author (Material S3)	Reach (how has screening been accounted for?)	Effectiveness (what are the reported outcomes?)	Adoption (where has the study been led?)	Implementation (was the screening process clearly reported? Any methodological challenges?)	Maintenance (sustainability, limitations, solutions proposed?)
Author, year	Target population–inclusion/exclusion–% screened vs. not screened	Mortality–Survival–Cancer stage–Interval cancers–Quality metrics	Country/region–Registry level (national, regional)–Setting	Screening method–Registry linkage–Bias adjustment–Missing data reported?	Long-term outcomes–Registry continuity–Challenges mentioned–Proposed improvements
Roberts *et al.* (1990)	Women aged 45–64 invited to annual screening. Compared screen-detected (prevalent and subsequent screens), interval, and non-screened cases.	Reported a 17% non-significant reduction in breast cancer mortality in the screened group.	Conducted in Edinburgh using a national cancer registry.	Two-view mammography; Women flagged at the General Register Office to track incidence and death; adjusted for SES (socioeconomic status).	Data lacked statistical power. Follow-up continuation was planned; authors indicated a future report after 12 years, suggesting intent to monitor sustainability, though long-term data were not yet available.
Autier *et al.* (2002)	Women aged 50–64 invited to biennial screening. Compared screen-detected, interval, and non-screened cases.	Screen-detected tumours were significantly smaller, earlier stage, and more often node-negative than clinically detected tumours.	Conducted in Luxembourg using a national cancer registry.	Two-view mammography; Cross-linking of Social Security Office records and the Mammography Programme to classify participants, adjusted for age.	National registry data showed good coverage, but inadequate linkage between the screening programme and the registry limited sustainability despite partial maintenance.
Garvican and Littlejohns (1998)	Women aged 50–64. Compared screen-detected, interval, eligible but not yet invited, non-attenders, and not registered.	Screen-detected cancers had a significantly better prognosis; women in this group were less deprived.	Conducted in England using a regional cancer registry.	Mammography details not clearly reported; manual matching using identifiers (surname, forename, date of birth, address); No adjustment variables.	Reported poor data coverage and missing prognostic variables before 1992. Matching limitations noted. Authors recommended wider use of new national health service numbers to improve matching and tracking.
Fracheboud *et al.* (1999)	Women aged 50–69 invited to biennial screening. Compared screen-detected, interval, and non-screened cases.	The tumour size distribution of interval cancers was less favourable than screen-detected cancers but more favourable than non-screened cancers.	Conducted in the Netherlands using a regional cancer registry.	Mammography; Linkage using date of birth, first four characters of surname and postal code; adjusted for age.	The study reported a 3-year delay in registry records and 100% data coverage by the national registry. Under-reporting of interval cancers occurred due to regional-level linkage.
Defossez *et al.* (2018)	Women aged 50–74 invited to biennial screening. Compared screen-detected, interval, and non-screened cases.	Screen-detected tumours were earlier stage, lower grade, with fewer hormone receptor–negative tumours and fewer distant metastases.	Conducted in France using a regional cancer registry.	Two-view mammography; Linkage of French screening registry and vital statistics to the cancer registry; adjusted for Tumour Node Metastasis (TNM) stage.	The study reported complete and robust data from the regional registry.
Cortesi *et al.* (2006)	Women aged 50–69 invited to biennial screening. Compared screen-detected, interval, and non-screened cases.	Screen-detected cancers were smaller, more likely node-negative, lower grade, had low proliferative activity and positive receptor status, and showed better 5-year survival.	Conducted in Italy using a regional cancer registry.	Two-view mammography; cross-linkage between screening programme and Modena Cancer Registry; adjusted for lead-time and length bias.	Authors recommended implementing cancer registries in all areas covered by screening programmes.
Caumo *et al.* (2010)	Women invited to screening. Compared screen-detected, interval, and non-screened cases.	Interval cancers had similar growth rate and presentation to non-screened cancers, while screen-detected cancers had more favourable features.	Conducted in Italy using a regional cancer registry.	Mammography; Linkage method not reported; No adjustment variables.	Complete data available in the regional registry.
Otto *et al.* (2010)	Not reported	Suggested a 52–56% reduction in breast cancer mortality among women invited and attending the screening programme.	Conducted in the Netherlands using a regional cancer registry.	Mammography; Linkage between the cancer registry and general practitioners’ records; No adjustment variables.	Not reported
Ganry *et al.* (2001)	Women aged 50–69 invited to screening. Compared screen-detected, interval, and non-screened cases.	Tumour size distribution of interval cancers was less favourable than screen-detected cancers and non-screen-detected cancers	Conducted in France using a regional cancer registry.	Mammography; Linkage by probability matching using coded surname, initials, date of birth and postcode; adjusted for age.	Reported 100% coverage and accurate match rate. Registry had a 3-year reporting delay.
Nagtegaal *et al.* (2010)	Women aged 50–69 invited to screening. Compared screen-detected cancers, interval cancers, and not recently exposed cancers (not yet invited then non-attenders).	Screen-detected cancers were smaller, better differentiated, and less likely to be node-positive, with substantially better survival.	Conducted in the UK using a regional cancer registry.	Mammography details not clearly reported; Linkage method not reported; adjusted for nodal status, tumour size, grade, tumour type, age, and year of diagnosis.	Not reported
Aarts *et al.* (2010)	Women aged 50–69 invited to biennial screening. Compared screen-detected, interval, and non-screened cases.	Survival was substantially better in screen-detected cancers. Among non-attendees and interval cancers, socioeconomic survival disparities were largely explained by stage and, to a lesser extent, therapy. Comorbidity explained most inequalities among screen-detected patients	Conducted in the Netherlands using a regional cancer registry.	Mammography; databases from Bevolkings Onderzoek Borstkanker Zuid (BoBZ) and the Eindhoven Cancer Registry linked; adjusted for age, stage, therapy, comorbidity, and combinations of these.	Reported good data coverage.
Bennett *et al.* (2006)	Women aged 50–64 invited to screening. Compared invited (screen-detected, interval, non-attender, lapsed attender) and uninvited groups.	Data quality was insufficient for a precise estimate of mortality reduction using surrogate measures.	Conducted in England using a national cancer registry.	Mammography; Linkage between cancer registries and screening unit; no adjustment variables.	Reported variable registry completeness, missing screening status, weak communication between registries and quality assurance centres, and lack of standardized data collection. Recommended improved registry training and collaboration.
Gill Lawrence *et al.* (2008)	Women aged 50–69 invited to triennial screening. Compared screen-detected, interval, and non-screened cases.	Women with screen-detected breast cancer had a substantial survival advantage over women with non-screened cancer.	Conducted in the UK using a regional cancer registry.	Two-view mammography; Linkage between (Office for National Statistics) ONS and cancer registries; adjusted for lead-time and length bias.	Not reported
Davies *et al.* (2013)	Women aged 50–64 invited to screening. Compared screen-detected, interval, and non-screened cases.	Screen-detected cancer incidence was lower among deprived women, whose 5-year survival was worse than affluent women; survival differences were smaller for screen-detected cancers.	Conducted in the UK using a regional cancer registry.	Mammography; Linkage between hospital records and National Health Service central register; adjusted for age and deprivation.	Poor screening-status coverage. Work underway with private providers to improve registry data capture.
Paajanen *et al.* (2006)	Women aged 50–59 invited to biennial screening. Compared screen-detected, interval, and non-screened cases.	Screen-detected cancers were smaller, diagnosed earlier, and had better 10-year survival (90% vs 70%) than cancers detected by other means.	Conducted in Finland using a national cancer registry.	Mammography; Linkage between the Finnish Cancer Registry and Mammographic Working group; No adjustment variables.	Not reported
Garvican and Littlejohns (1996)	Women aged 50–64 invited to triennial screening. Compared screen-detected cancers, interval cancers, Non-attender, Uninvited, and Not registered groups.	Breast screening programme appeared to have low impact on overall breast cancer diagnosis in South East Thames,	Conducted in the UK using a regional cancer registry.	Mammography; Linkage using name, previous surname and address; No adjustment variables.	Estimated 2–3 years to achieve data completeness. Matching was labour-intensive, time-consuming and incomplete, with poor patient coding reported.
de Munck *et al.* (2020)	Women aged 49–74 invited to triennial screening. Compared screen-detected, interval, and non-screened cases.	Screen-detected cancers were less often diagnosed at advanced stage.	Conducted in the Netherlands using a national cancer registry.	Mammography; Linkage between NCR and Netherlands breast cancer screening registry; adjusted for age at diagnosis, year and Socioeconomic status (SES).	Reported good data coverage.
O’Brien *et al.* (2018)	Women aged 50–64 invited to biennial screening. Compared screen-detected cancers, interval cancers, lapsed attenders and non-participants.	No significant survival difference was observed for screen-detected cancers in the 5-year post-diagnosis period.	Conducted in Ireland using a national cancer registry.	Two-view mammography; Linkage of screening records, NCRI data, cancer registrations and death certificates; adjusted for age, marital status, smoking, deprivation, co-morbidities, tumour subtype, stage and grade.	Reported good data coverage.
Threlfall *et al.* (2003)	Women aged 54 years or younger invited to screening. Compared attenders (first-screen cancers, rescreening cancers, Interval cancers, lapsed-attender cancers) and non-participants.	No specific outcomes reported; assumed comparative analysis across categories.	Conducted in England using a regional cancer registry.	Mammography details not clearly reported; Linkage of screening histories with NHS central register; No adjustment variables.	Reported good data coverage. Linkage relied on administrative systems without quality-control detail.
Fracheboud *et al.* (2001)	Women aged 50–69 invited to biennial screening. Compared screen-detected, interval, and non-screened cases.	Stage distribution of screen-detected cancers was more favourable than interval cancers and unscreened cancers.	Conducted in the Netherlands using a regional cancer registry.	Two-view mammography; Linkage of regional screening files and cancer registries; No adjustment variables.	Not reported
Paci (2007)	Women aged 50–69 invited to biennial screening. Compared invited (prevalent screen-detected, repeated screen-detected, non-responders) and non-invited, screen-detected and unscreened groups.	Women invited had a greater significant mortality reduction	Conducted in Italy using a regional cancer registry.	Two-view mammography; Linkage method not reported; No adjustment variables.	Not reported
Musolino *et al.* (2012)	Women aged 50–69 invited to biennial screening. Compared screen-detected, interval, and non-screened cases.	Tumours with high grade, high proliferative rate, negative oestrogen receptor status or HER2-positive status were more likely diagnosed in the interval between screens.	Conducted in Italy using a regional cancer registry.	Two-view mammography; Linkage between Parma cancer registry and service database using first name, surname, date of birth and date of last mammography; adjusted for age and tumour size.	Reported good data coverage with verification by a reference pathologist.
Bulliard *et al.* (2009)	Women aged 50–69 invited to biennial screening. Compared screen-detected, interval, and non-screened cases.	Mammography screening strongly influenced breast cancer stage distribution in Switzerland	Conducted in Switzerland using a regional cancer registry.	Two-view mammography; Linkage screening programme records and population-based cancer registries; No adjustment variables.	Not reported
Braun *et al.* (2018)	Women aged 50–69 invited to biennial screening. Compared screen-detected, interval, and non-screened cases.	Screening participants with invasive cancer generally required less intensive surgical and systemic therapy than non-participants, including when interval cancers were considered.	Conducted in Germany using a regional cancer registry.	Mammography details not clearly reported; Linkage method not reported; No adjustment variables.	Reported 95% registry data coverage .
Poiseuil *et al.* (2019)	Women aged 50–74 invited to biennial screening. Compared screen-detected cancers, interval cancers, lapsed attenders and non-participants	Survival rates were lower for non-attenders than for women with screen-detected cancers	Conducted in France using a regional cancer registry.	Two-view mammography; Linkage between AGIDECA (Association Girondine pour le Dépistage des Cancers) and registry data; adjusted for lead-time.	Reported registries as reliable and exhaustive data sources.
De Munck *et al.* (2016)	Women aged 50–75 invited to screening. Compared screen-detected, interval, and non-screened cases.	Screen-detected cancers were less often advanced stage than interval cancers and non-screen-related cancers	Conducted in the Netherlands using a national cancer registry.	Mammography details not clearly reported; Linkage between registry data and Dutch national breast cancer screening programme; No adjustment variables.	Not reported

## Results

### General characteristics of the included publications

**Figure 2.**
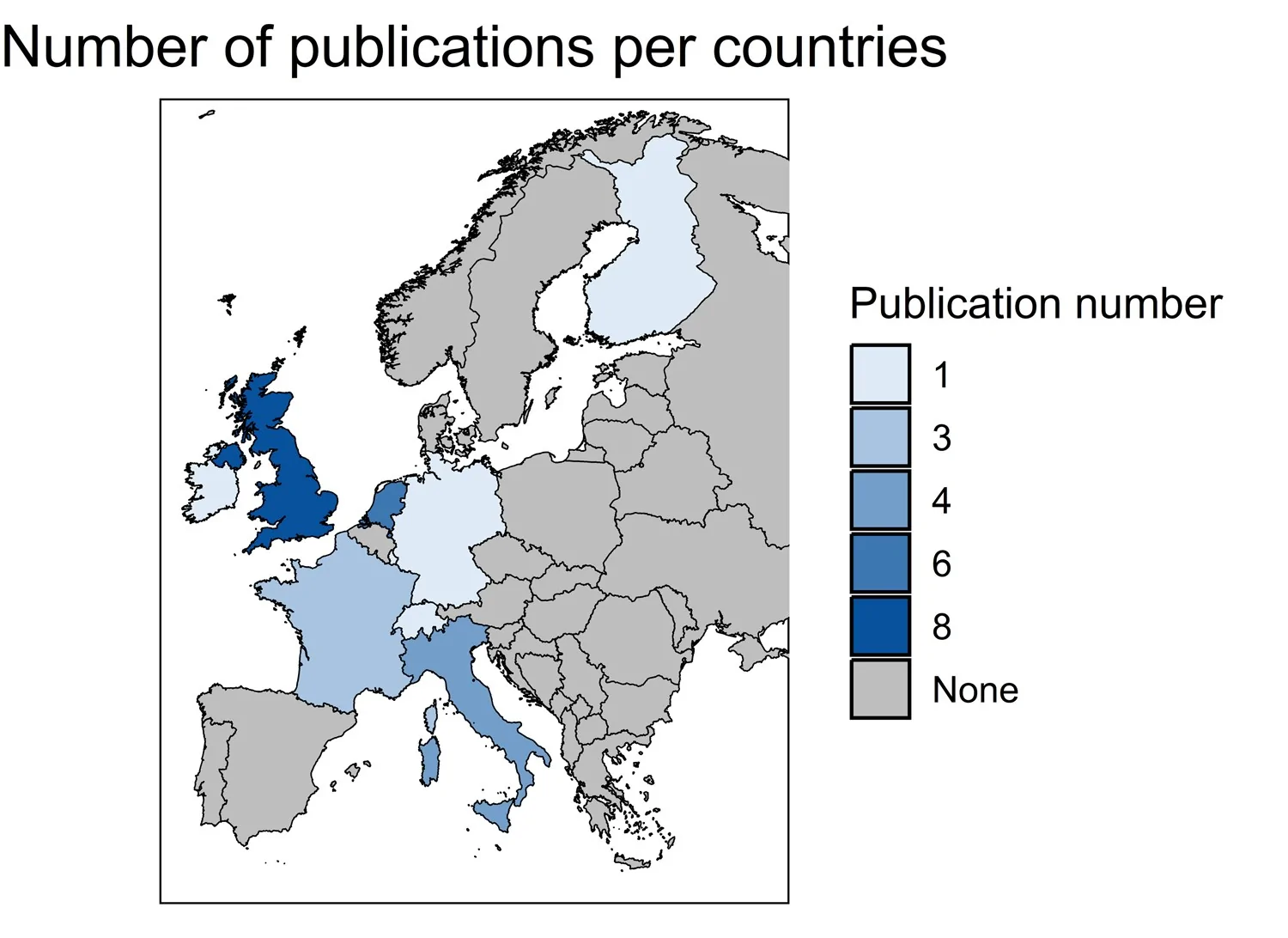
Number of included publications per country.

Twenty-six studies published between 1990 and 2020 were included. Most studies originated from the UK (*n* = 8) and were retrospective cohort studies (Fig. 2). One study (*n* = 1) each used a randomized controlled trial and case-control design, reflecting methodological diversity.

### Use of screening and registry data for programme evaluation

#### Screening programme data

All studies evaluated organized breast cancer screening programmes implemented in Europe between 1979 and 2015. Mammography was the primary screening modality, with several studies specifying two-view mammography. Target populations generally included individuals at average risk aged 50–69 years, although some programmes used slightly different age ranges.

Screening intervals varied among the studies. Biennial screening was most prevalent. Triennial intervals were noted in three studies, and annual screening was reported in one study. Eight studies did not report screening frequency.

#### Registry data sources and outcomes

PBCRs, together with data from screening programmes, were used to identify and classify breast cancer cases. Registry data covered the years 1979–2017. In most studies, the PBCR data aligned with the study period; however, seven studies extended the timeframe to accommodate delays in updating screening information. Seven registries were national, and 19 were regional. Primary registry-derived outcomes included cancer incidence, stage, tumour size, grade, and treatment. Additional outcomes included mortality, survival, oestrogen and progesterone receptor status, nodal involvement, HER2 expression, histology, proliferation rate, surgery type, invasive status, and comorbidities.

#### Data linkage and classification by detection mode

Datasets on the study populations were generally created through pseudo-anonymized linkage between screening data sources and PBCRs. Four studies did not report their linkage method. Case identification was performed by matching key identifiers such as date of birth, address, name, or initials. Most studies used deterministic linkage based on unique personal identifiers, while older or regionally focused studies used probabilistic matching based on non-unique identifiers such as name and address. This linkage enabled the classification of individuals into screen-detected, interval, and non-screened cancer groups according to detection pathway.

Breast cancer cases categorized as screen-detected cancers, were those identified during scheduled screening; interval cancers, were diagnosed after a negative mammogram and before the next screening round; and non-screened cancers, were cancers occurring in individuals who were not invited or did not participate in screening. Some studies further distinguished initial and subsequent screening rounds within screen-detected cases.

### Indicators used to evaluate screening programmes

#### Screening quality indicators

Several studies used cancer registry data to assess the quality and performance of breast cancer screening programmes through indicators such as detection rates, interval cancer rates, stage distribution at diagnosis, and incidence-based mortality (Table 2). These indicators are commonly used to evaluate screening effectiveness and programme performance.

**Table 2.** Overall tendencies of outcomes reported across studies

Outcome	*N* studies	Indicators	Overall trend	Direction of effect	Implications
Mortality	3	RR, OR, incidence-based mortality	17%–56% lower mortality among screened women; often non-significant once adjusted	Screen-detected < interval < non-screened	Highlights the need to correct for selection, lead-time, length-time bias
Survival	9	OS, BCSS, relative survival, net survival	Higher survival for screen-detected cancers; differences shrink after bias correction	Screen-detected > Interval > non-screened	Differences partly explained by stage distribution
Clinical and pathological features	9	TNM stage, size, nodal status, grade, biomarkers	Earlier stage, smaller tumours, fewer positive nodes among screen-detected	Screen-detected > Interval and non-screened	Strong consistency across countries
Screening performance indicators	5	Detection rate, interval cancer rate, sensitivity, stage at diagnosis	Interval cancer rates and detection rates generally within expected ranges; heterogeneity across settings	Variable but overall acceptable	Reinforces need for harmonized definitions

Early evaluations demonstrated that cancer registry data could support monitoring of key screening indicators. For example, studies in the Netherlands and the UK assessed programme sensitivity, interval cancer rates, and stage distribution as measures of screening quality. Interval cancer rates reported by Fracheboud *et al.* (Material S3) were ∼0.95 per 1000 woman-years during the 2 years following screening, with screen-detected cancers presenting with more favourable tumour characteristics than interval or non-screened cancers. Similarly, Threlfall *et al.* (Material S3) reported that regions with higher screening participation had lower rates of advanced-stage disease despite slightly higher overall invasive cancer incidence, suggesting earlier detection through screening (Table 1).

Other studies examined the role of cancer registries in ensuring accurate programme monitoring. Bennett *et al.* (Material S3) evaluated the completeness and reliability of registry data for identifying screen-detected, interval and symptomatic cancers in the UK. Their findings highlighted that incomplete or inconsistent registry reporting could limit the ability to estimate screening outcomes reliably, particularly when surrogate indicators are used to infer mortality reduction.

These findings underline the importance of high-quality cancer registry data for evaluating screening performance. Reliable linkage between screening programmes and population-based cancer registries is essential to accurately identify detection modes and calculate key indicators such as interval cancer rates and stage distribution. In Europe, such indicators are recommended within the quality assurance framework established by the European Commission for organized breast cancer screening programmes.

Overall, the studies reviewed suggest that PBCR data can provide valuable indicators for monitoring screening performance. However, variations in data completeness, linkage practices, and indicator definitions may limit the comparability of programme evaluations across settings.

#### Pathological and clinical characteristics

Several studies examined tumour characteristics according to detection mode, including tumour size, stage at diagnosis, lymph node involvement, histological grade, and biological markers. Overall, screen-detected cancers were consistently associated with more favourable clinical and pathological profiles, whereas interval and non-screened cancers more frequently presented with advanced or aggressive disease (Table 2).

Across European settings including Luxembourg, The Netherlands, and Switzerland, screen-detected cancers were significantly smaller and diagnosed at earlier stages than interval or non-screened cancers. For example, the proportion of tumours larger than 20 mm was substantially higher among unscreened women than among screen-detected cases, while advanced stage disease was markedly less frequent in screen-detected cancers than in interval or non-screened cancers. Similar patterns were observed for lymph node involvement and tumour size, with interval and non-screened cancers showing higher proportions of node-positive and larger tumours.

Differences in biological characteristics were also reported. Interval cancers were more likely to exhibit unfavourable tumour profiles, including higher histological grade, greater proliferative activity, and hormone-receptor-negative or HER2-positive status. However, comparisons between interval and non-screened cancers were not always statistically significant across studies.

Studies comparing screening participants and non-participants also reported more favourable tumour characteristics among participants, including higher frequencies of ductal carcinoma *in situ*, earlier tumour stage, lower rates of high-grade tumours, and fewer triple-negative cancers. These differences were reflected in treatment patterns, with screening participants more likely to undergo breast-conserving surgery and less likely to require systemic chemotherapy.

Overall, the evidence indicates that screen-detected cancers tend to be diagnosed earlier and present with more favourable pathological features, whereas interval cancers often display more aggressive characteristics and resemble symptomatic cancers. However, none of the studies adjusted for potential biases such as lead-time, length-time, or self-selection bias.

#### Survival

Several studies assessed overall survival (OS) or breast cancer–specific survival (BCSS) according to detection mode (Table 2). Across studies, screen-detected cancers consistently showed better survival than interval or non-screen-detected cancers.

Five-year OS for screen-detected cancers ranged from 91% to 94%, compared with 77%–84% for interval or non-screened cancers. Similar patterns were observed for 10-year survival, with estimates of 86%–90% for screen-detected cancers and 58%–70% for interval or symptomatic cancers. Breast cancer–specific survival showed comparable trends, with 5-year BCSS above 93% for screen-detected cancers and lower estimates among interval cancers and non-attendees.

Some studies also reported socioeconomic differences, with lower survival among more deprived populations, particularly for non-screen-detected cancers.

However, several analyses indicated that survival differences were partly explained by tumour characteristics and stage at diagnosis. Adjustments for factors such as tumour size, nodal status, grade, subtype, and socioeconomic variables substantially reduced or eliminated the observed survival advantage associated with screen detection in some studies.

#### Mortality

Three studies evaluated breast cancer–specific or overall mortality according to detection mode (Table 2). Overall, screen-detected cancers were associated with lower mortality compared with non-screened or interval cancers.

Mortality reductions among screened women ranged from 17% to 56%, although statistical significance varied across studies. One study reported significantly lower mortality among screened women in the southwest Netherlands, while studies conducted in Edinburgh and Italy reported non-significant reductions. Another study comparing regions with different screening participation levels observed lower mortality in the area with higher screening uptake.

Few studies reported mortality separately for interval cancers. Available evidence suggests that their prognosis lies between that of screen-detected and non-screened cancers, although comparisons remain limited due to inconsistent definitions, sparse reporting, and adjustments for potential biases [12].

### Methodological gaps across studies

Several cross-cutting methodological limitations were identified. Definitions of detection mode, particularly interval cancers, varied across studies. Reporting of linkage procedures and screening indicators was inconsistent. Only a minority of studies adjusted for major screening-related biases such as lead-time, length-time, or self-selection bias. In addition, most included studies were published before 2010, indicating limited recent peer-reviewed evaluations despite the continued maturity of organized screening programmes in Europe.

## Discussion

This scoping review identified 26 studies that used population-based cancer registry (PBCR) data to evaluate organized breast cancer screening programmes in Europe. Across these studies, the evidence shows that PBCRs are valuable tools for programme evaluation because they enable population-level follow-up, linkage of screening exposure with cancer outcomes, and comparisons between screen-detected, interval, and non-screened cancers. However, despite this clear potential, the literature was characterized by substantial methodological heterogeneity that limits comparability across studies and reduces the policy value of available evidence. Taken together, these findings suggest that the main limitation of current evidence is not the lack of data, but the lack of harmonized analytical approaches to evaluate screening effectiveness across European settings.

In this context, most included studies used retrospective registry-based cohort designs. While these designs provide large population coverage and minimize selection bias compared with some prospective cohorts, they remain subject to important methodological limitations. In particular, registry-based analyses may lack detailed screening histories or clinical variables needed to evaluate outcomes such as overdiagnosis, and they rely on accurate linkage between screening programmes and cancer registries. These limitations should therefore be considered when interpreting results derived from PBCR-based cohort studies.

Despite methodological differences, a consistent finding across included studies was that screen-detected cancers were associated with more favourable outcomes than interval or non-screened cancers. Screen-detected cases were generally diagnosed at earlier stages, had smaller tumour size, lower nodal involvement, and better survival estimates. By contrast, interval cancers more frequently showed adverse pathological features and outcomes closer to symptomatic cancers. These patterns were observed across multiple settings and are consistent with the expected effects of earlier diagnosis through organized screening. In addition, some studies reported lower rates of advanced disease or lower mortality in populations with higher screening participation, suggesting potential programme benefit at population level.

However, interpretation of these findings requires caution. The most consequential limitations appear to be the inconsistent definition of interval cancers, the limited adjustment for self-selection bias, and the lack of transparent reporting of data linkage procedures, as these directly affect the validity and comparability of screening effectiveness estimates. First, a major finding was the lack of harmonization in core definitions and reporting practices. Differences were observed in definitions of detection mode, particularly for interval cancers, whose definitions were inconsistent across studies. Secondly, only a minority of studies adjusted for major screening-related biases such as lead-time bias, length-time bias, or self-selection bias, highlighting an important methodological gap. Survival comparisons between detection modes may be influenced by screening-related biases, particularly lead-time bias, length-time bias and overdiagnosis [12]. The survival advantages among screen-detected cancers may partly reflect earlier diagnosis without delaying death, preferential detection of slower-growing tumours, or differences between women who do and do not participate in screening. Also, overdiagnosis can artificially inflate survival among screen-detected cancers because indolent tumours that would not have become clinically relevant are more likely to be detected through screening. Several studies showed that adjustment for tumour stage, biological characteristics, or socioeconomic factors substantially attenuated observed differences. Consequently, the direction of association was relatively consistent, but the true size of benefit was less certain.

Finally, although breast cancer screening in Europe is guided by the European quality assurance framework, implementation of screening programmes varies across countries with respect to eligibility criteria, screening intervals, invitation systems, and levels of opportunistic screening. Linkage procedures between screening datasets and PBCRs were often insufficiently described, and some studies did not report whether deterministic or probabilistic linkage methods were used. Reporting of quality indicators such as interval cancer rates, sensitivity, specificity, recall rates, or participation also varied considerably. These differences complicate cross-country comparisons and weakens the ability to benchmark programme performance across Europe.

European screening programmes are guided by the recommendations of the European Commission through the European Guidelines for Quality Assurance in Breast Cancer Screening and Diagnosis. These guidelines include defined performance indicators and quality assurance metrics for programme evaluation. More recent European quality assurance guidance also provides detailed indicators related to screening performance, data completeness, and programme monitoring [13]. However, their uptake in peer-reviewed evaluations appears inconsistent. Stronger alignment between programme monitoring systems, registry outputs, and research reporting standards would improve comparability and policy translation.

Another important observation was the temporal distribution of included studies. Most were published before 2010, despite the continued maturity of organized screening programmes and cancer registries in Europe. This should not necessarily be interpreted as a lack of recent evaluation activity. Many mature programmes now disseminate monitoring results through national reports, quality assurance documents, or internal governance mechanisms rather than peer-reviewed journals. In addition, increasing data-governance requirements and legal constraints may have complicated linkage-based research. More recent academic studies may also focus on narrower themes such as risk stratification, imaging technology, inequalities, or treatment pathways rather than broad programme evaluation. Nonetheless, the limited visibility of recent peer-reviewed registry-based evaluations creates an evidence gap for comparative public health learning across countries.

This review also highlights the strategic importance of PBCRs beyond screening effectiveness alone. Registry-linked data can support evaluation of equity in access, regional variation, deprivation-related inequalities, treatment patterns, and long-term outcomes. As screening programmes evolve toward more risk-adapted and personalized approaches, robust registry infrastructures will become increasingly important for real-world monitoring of benefits, harms, and unintended consequences.

Several limitations of this review should be acknowledged. By focusing on peer-reviewed publications, the analysis may not fully capture the breadth of programme evaluation activities conducted within European screening systems. Many organized screening programmes produce annual monitoring reports or quality assurance documents that are not published in scientific journals. These grey literature sources frequently include PBCR-based indicators and may follow standardized European performance metrics more closely than research articles. Their exclusion may therefore lead to an underrepresentation of recent programme evaluations and of screening systems with well-developed internal monitoring structures.

Another methodological consideration relates to the literature search strategy. Identifying studies that explicitly used PBCR data proved challenging because references to cancer registries are often reported only in the methods section rather than in titles or abstracts. Consequently, some relevant studies may not have been retrieved if registry linkage was not clearly described in searchable fields. This limitation may partly explain why certain studies from Nordic countries, which are known for their high-quality registry infrastructure and deterministic linkage systems, were not included despite the strong tradition of registry-based screening research in these settings. Finally, as a scoping review, the objective was to map evidence rather than formally assess risk of bias or pool effect estimates.

Nonetheless, the studies included in this review demonstrate the substantial value of PBCRs for evaluating cancer screening programmes at the population level. The findings highlight both the potential and the current limitations of PBCR-based evaluations in Europe. While high-quality registry infrastructures are widely available, their analytical use remains insufficiently standardized. In several European countries, high-quality PBCR systems allow comprehensive evaluation of screening programmes when combined with screening registries and administrative data sources [14, 15].

Beyond Europe, several regions have implemented integrated screening surveillance systems that demonstrate the potential of structured registry linkage. For example, the Breast Cancer Surveillance Consortium (BCSC) in the USA links mammography screening data with cancer registries to generate standardized indicators including interval cancer rates, screening sensitivity, and long-term outcomes [16–18]. Similarly, national screening monitoring systems in Canada and Australia provide regular programme evaluation based on standardized data collection and reporting frameworks [19–21]. Compared with these systems, European evaluations appear less standardized and less consistently reported in the peer-reviewed literature, despite the availability of comparable registry infrastructures.

Overall, although registry-based analyses offer valuable population-level insights, greater methodological harmonization is needed to improve comparability across studies. This includes clearer definitions of detection modes, consistent reporting of screening indicators and more systematic use of bias-adjustment methods.

Several analytic approaches have been proposed to address screening-related biases in observational data. Methods such as self-selection correction, modelling of lead-time effects, and use of incidence-based mortality can improve the validity of screening evaluations. Wider adoption of such approaches in registry-based studies would strengthen the interpretation of screening outcomes.

Recent European initiatives aim to improve harmonization and data integration across screening programmes. Groupe pour l‘Épidémiologie et l‘Enregistrement du Cancer dans les Pays de Langue Latine (GRELL) and the International Agency for Research on Cancer Global Initiative for Cancer Registry Development (IARC GICR) have supported collaboration and standardization among cancer registries, yet some countries still do not report breast cancer screening data systematically [22–24]. Projects such as the European Cancer Information System and the EUCanScreen Joint Action are working to develop standardized indicators and strengthen the infrastructure for monitoring cancer screening programmes across Member States [14, 15]. Such initiatives may facilitate more comparable and transparent evaluation of screening performance in the future.

In conclusion, this review provides a structured overview of how PBCR data have been used to evaluate organized breast cancer screening programmes in Europe. The main challenge identified is no longer the absence of data systems, but the absence of sufficiently harmonized analytical frameworks to maximize their value. Future European evaluations would benefit from standardized definitions of detection mode, transparent linkage reporting, routine correction for major screening biases, and a core outcome set including effectiveness, harms, and equity indicators. Strengthening these elements would improve comparability between programmes and support more accountable, evidence-informed cancer screening policy across Europe.

## Supplementary Material

ckag090_Supplementary_Data

## Data Availability

The data underlying this article are available in the article and in its online supplementary material.
