# Correction to: Use of population-based cancer registry data to evaluate organized breast cancer screening programmes in Europe by mode of detection: a scoping review

**DOI:** 10.1093/eurpub/ckag189

**Published:** 2026-11-15

This is a correction to: Wendy Kam, Elodie Guillaume, Quentin Rollet, Dafina Petrova, Nicolás Francisco Fernández-Martínez, Koen van Herck, Marià Carulla, Claudia Pla, Francina Saladié, Cristina Miracle, Vesna Zadnik, Sonja Tomšič, Anne Cowppli-Bony, Guy Launoy, Claudine Backes, Use of population-based cancer registry data to evaluate organized breast cancer screening programmes in Europe by mode of detection: a scoping review, *European Journal of Public Health*, Volume 36, Issue 4, August 2026, ckag090, https://doi.org/10.1093/eurpub/ckag090

In the originally published version of this manuscript, co-author Marià Carulla was erroneously listed as affiliated to “Department of Epidemiology, Regional Health Council, IMIB-Arrixaca, Murcia University, Murcia, Spain”, and co-author Cristina Miracle was erroneously listed as affiliated to “Hospitalet de Llobregat, Catalan Institute of Oncology (ICO), Cataluna, Espana”.

The correct affiliation for both authors is “Cancer Epidemiology and Prevention Service, Hospital Universitari Sant Joan de Reus, IISPV, Reus, Spain”.

This correction has been made to the article, and all subsequent affiliations have been re-numbered accordingly.

