# Supplementary material for: Use of population-based cancer registry data to evaluate organized breast cancer screening programmes in Europe by mode of detection: a scoping review
Source: Eur J Public Health. 2026 Jun 17;36(4):ckag090. doi: 10.1093/eurpub/ckag090 (PMC13275118; doi:10.1093/eurpub/ckag090)
Supplement: ckag090_Supplementary_Data [file ckag090_supplementary_data.zip › ejph-2026-03-om-0248-File004.docx]

**SCREENIT - Protocol of the scoping review**

Quentin Rollet, Claudine Backes, Koen Van Herck, Dafina Petrova

Research question:

Assessing the effectiveness of cancer screening programmes in Europe using population-based cancer registries: a scoping review of the breadth and depth of what has already been done.

Research algorithm:

For the scoping review PubMed and EMBASE databases will be interrogated by using the below suggested algorithms. These algorithms will be run by localisation, starting with breast cancer, then colorectal cancer and finally cervical cancer.

1. **PubMed algorithm suggested**:

**Breast cancer: *((("cancer registry"[TW] OR "cancer registries"[TW]) OR (Registries[MH] AND (Neoplasms[MH] OR cancer[TW] OR cancers[TW]))) AND (Breast Neoplasms [MH] OR "Breast Cancer"[TW])) AND (Mass Screening[MH] OR "Screening Program*"[TW] OR "Cancer screening*"[TW])***

- On 03 March 2023*,* PubMed research returned 1,020 articles.

**Colorectal cancer*: ((("cancer registry"[TW] OR "cancer registries"[TW]) OR (Registries[MH] AND (Neoplasms [MH] OR cancer[TW] OR cancers[TW]))) AND ("Colorectal Neoplasms"[MH] OR bowel[TW] OR colorect*[TW])) AND (Mass Screening[MH] OR "Screening Program*"[TW] OR "Cancer screening*"[TW])***

**Cervical cancer: *((("cancer registry"[TW] OR "cancer registries"[TW]) OR (Registries[MH] AND (Neoplasms [MH] OR cancer[TW] OR cancers[TW]))) AND ("Uterine Cervical Neoplasms"[MH] OR cervi*[TW])) AND (Mass Screening[MH] OR " Screening Program*"[TW] OR "Cancer screening*"[TW])***

1. **EMBASE algorithm suggested:**

**Breast cancer:** *(exp mass screening/ or mass screening.ti,ab. or screening program$.ti,ab. or cancer screening$.ti,ab.) and ((exp cancer registry/ or cancer registr$.ti,ab.) and (exp breast tumor/ or breast cancer.ti,ab)) and limit 26 to "remove medline records"*

- On 03 March 2023*,* Embase research returned 490 articles.

**Colorectal cancer*:*** *(exp mass screening/ or mass screening.ti,ab. or screening program$.ti,ab. or cancer screening$.ti,ab.) and ((exp cancer registry/ or cancer registr$.ti,ab.) and (exp* ***colorectal cancer*** *or* ***colorectal cancer****.ti,ab or* ***bowel.ti,ab or colorectal.ti,ab*** *)) and limit 26 to "remove medline records"*

**Cervical cancer:** *(exp mass screening/ or mass screening.ti,ab. or screening program$.ti,ab. or cancer screening$.ti,ab.) and ((exp cancer registry/ or cancer registr$.ti,ab.) and (exp* ***uterus cancer*** *or exp* ***'uterine cervix cancer or cervi$.ti,ab****)) and limit 26 to "remove medline records"*

Scoping review – suggested screening process:

- Volunteers will be recruited and grouped in teams of researchers (= team of researchers)
- Title and abstracts will be read and evaluated by at least (depending on the language and availability) two researchers
- An initial test will be carried out on a few articles to ensure the relevance of the exclusion criteria and the homogeneity of the decisions.

**Exclusion method**

Table 1: Exclusion criteria - scoping review – SCREENIT

**Exclusion criteria**

| *For all articles* | |
| --- | --- |
| 1 | Study outside of the European continent (Table 2) |
| 2 | No measure of cancer screening intensity (either individual, aggregated, or proxies) |
| 3 | No analysis of PBCR outcome (with PBCR outcomes defined as any information from the PBCR regarding incidence, progression of the disease at diagnosis, treatment, follow-up, and vital status) |
| 4 | No cancer screening programme (with ‘screening programme’ defined as a public health policy specifying at least one screening exam, a screening interval, and an eligible population) |
| 5 | Other localisations than breast cancer |
| 6 | Simulation/Methodological/theoretical/interventional paper/Not original research |
| 7 | No link between a screening intensity measure and a PBCR outcome |
| 8 | No evaluation on the eligible population (defined by the article) |
| *For articles set aside for which no help could be found* | |
| 9 | Beyond the language skill available to the group of authors |

* As we are stratifying the work by cancer localisation: when working on breast cancer, colorectal & cervical cancer are excluded, when working on colorectal cancer, breast & cervical cancer are excluded, and when working on cervical cancer, breast & colorectal cancer are excluded.

Reviewers should select only one exclusion criterion per article, the higher they find in Table 1 below. This criterion must be filled in the box “Reason” of Rayyan. In addition, we asked the authors to report only the number of the exclusion criteria.

Regarding article in a language different from the skills of the reviewers’ team, we ask to flag them – these cases will be dealt with later. To do so, we ask to use the box “Label” in Rayyan. We also propose that this box is used whenever the author feel it might be appropriate to highlight an article or to discuss some elements (e.g., “Article of interest”, “Maybe”, or “Language”).

Scoping review - Data extraction process:

Extraction phase will be conducted in three phases.

1. To ensure homogeneity between extractions and the relevance of the suggested extraction grid, all will extract few articles randomly chosen.
2. All remaining articles will be randomly assigned to the different teams of researchers and independently extracted. An independent researcher will be in charge to compare extractions. Exclusion criteria are available in Table 1.
3. Snowballing method: References of the eligible article should be studied, and relevant references kept. They will go through a new screening process before being potentially included. This step will stop at saturation or after 3 rounds.

Cases of disagreements:

Disagreements or doubts related to extractions and further exclusions of articles will be discussed in a larger group of co-authors. In case of non-agreement, someone will be chosen to impose a final decision.

Table 2 List of countries included in the European continent

| **List of countries in the European continent*** |
| --- |
| Albania |
| Andorra |
| Austria |
| Belarus |
| Belgium |
| Bosnia and Herzegovina |
| Bulgaria |
| Croatia |
| Czech Republic (Czechia) |
| Denmark |
| Estonia |
| Finland |
| France |
| Germany |
| Greece |
| Holy See |
| Hungary |
| Iceland |
| Ireland |
| Italy |
| Latvia |
| Liechtenstein |
| Lithuania |
| Luxembourg |
| Malta |
| Moldova |
| Monaco |
| Montenegro |
| Netherlands |
| North Macedonia |
| Norway |
| Poland |
| Portugal |
| Romania |
| Russia |
| San Marino |
| Serbia |
| Slovakia |
| Slovenia |
| Spain |
| Sweden |
| Switzerland |
| Ukraine |
| United Kingdom |

* https://www.worldometers.info/geography/how-many-countries-in-europe/
