# Supplementary material for: Use of population-based cancer registry data to evaluate organized breast cancer screening programmes in Europe by mode of detection: a scoping review
Source: Eur J Public Health. 2026 Jun 17;36(4):ckag090. doi: 10.1093/eurpub/ckag090 (PMC13275118; doi:10.1093/eurpub/ckag090)
Supplement: ckag090_Supplementary_Data [file ckag090_supplementary_data.zip › ejph-2026-03-om-0248-File006.docx]

**References of selected articles**

1. Roberts MM, Alexander FE, Anderson TJ, Chetty U, Donnan PT, Forrest P, et al. Edinburgh trial of screening for breast cancer: mortality at seven years. Lancet. 1990;335:241–6. <https://doi.org/10.1016/0140-6736(90)90066-E>.
2. Autier P, Shannoun F, Scharpantgen A, Lux C, Back C, Severi G, et al. A breast cancer screening programme operating in a liberal health care system: the Luxembourg Mammography Programme, 1992–1997. Int J Cancer. 2002;97:828–32. <https://doi.org/10.1002/ijc.10161>.
3. Garvican L, Littlejohns P. Comparison of prognostic and socio-economic factors in screen-detected and symptomatic cases of breast cancer. Public Health. 1998;112:15–20. <https://doi.org/10.1038/sj.ph.1900421>.
4. Fracheboud J, de de Koning HJ, Beemsterboer PMM, Boer R, Verbeek ALM, Hendriks JHCL, et al. Interval cancers in the Dutch breast cancer screening programme. Br J Cancer. 1999;81:912–7. <https://doi.org/10.1038/sj.bjc.6690786>.
5. Defossez G, Quillet A, Ingrand P. Aggressive primary treatments with favourable 5-year survival for screen-interval breast cancers. BMC Cancer. 2018;18:393. <https://doi.org/10.1186/s12885-018-4319-4>.
6. Cortesi L, Chiuri VE, Ruscelli S, Bellelli V, Negri R, Rashid I, et al. Prognosis of screen-detected breast cancers: results of a population based study. BMC Cancer. 2006;6:17. <https://doi.org/10.1186/1471-2407-6-17>.
7. Caumo F, Vecchiato F, Strabbioli M, Zorzi M, Baracco S, Ciatto S. Interval cancers in breast cancer screening: comparison of stage and biological characteristics with screen-detected cancers or incident cancers in the absence of screening. Tumori. 2010;96:198–201. <https://doi.org/10.1177/030089161009600203>.
8. Otto S, Boer R, Broeders MJM, Fracheboud J, Reijerink-Verheij J, Otten JDM, et al. 1N Evaluation of the breast cancer screening programme in Southwest Netherlands: a case–control study. Eur J Cancer Suppl. 2010;8:53. <https://doi.org/10.1016/S1359-6349(10)70028-8>.
9. Ganry OF, Peng J, Raverdy NL, Dubreuil AR. Interval cancers in a French breast cancer-screening programme (Somme Department). Eur J Cancer Prev. 2001;10:269–74. <https://doi.org/10.1097/00008469-200106000-00011>.
10. Nagtegaal ID, Allgood PC, Duffy SW, Kearins O, Sullivan EO, Tappenden N, et al. Prognosis and pathology of screen-detected carcinomas: how different are they? Cancer. 2011;117:1360–8. <https://doi.org/10.1002/cncr.25613>.
11. Aarts MJ, Voogd AC, Duijm LEM, Coebergh JWW, Louwman WJ. Socioeconomic inequalities in attending the mass screening for breast cancer in the south of the Netherlands – associations with stage at diagnosis and survival. Breast Cancer Res Treat. 2011;128:517–25. <https://doi.org/10.1007/s10549-011-1363-z>.
12. Bennett RL, Blanks RG, Moss SM, Roche MF, NHS Breast Screening Programme Evaluation Group. The effect of data quality at the time of introduction of population-based screening on the estimate of programme impact using surrogate outcome measures. J Med Screen. 2006;13:197–200. <https://doi.org/10.1177/096914130601300407>.
13. Lawrence G, Wallis M, Allgood P, Nagtegaal ID, Warwick J, Cafferty FH, et al. Population estimates of survival in women with screen-detected and symptomatic breast cancer taking account of lead time and length bias. Breast Cancer Res Treat. 2009;116:179–85. <https://doi.org/10.1007/s10549-008-0100-8>.
14. Davies EA, Renshaw C, Dixon S, Møller H, Coupland VH. Socioeconomic and ethnic inequalities in screen-detected breast cancer in London. J Public Health (Oxf). 2013;35:607–15. <https://doi.org/10.1093/pubmed/fdt002>.
15. Paajanen H, Kyhälä L, Varjo R, Rantala S. Effect of screening mammography on the surgery of breast cancer in Finland: A population-based analysis during the years 1985–2004. The American Surgeon™. 2006;72:167–71. <https://doi.org/10.1177/000313480607200215>.
16. Garvican L, Littlejohns P. An evaluation of the prevalent round of the breast screening programme in south east Thames, 1988–1993: achievement of quality standards and population impact. J Med Screen. 1996;3:123–8. <https://doi.org/10.1177/096914139600300304>.
17. de Munck L, Siesling S, Fracheboud J, den Heeten GJ, Broeders MJM, de Bock GH. Impact of mammographic screening and advanced cancer definition on the percentage of advanced-stage cancers in a steady-state breast screening programme in the Netherlands. Br J Cancer. 2020;123:1191–7. <https://doi.org/10.1038/s41416-020-0968-6>.
18. O’Brien KM, Mooney T, Fitzpatrick P, Sharp L. Screening status, tumour subtype, and breast cancer survival: a national population-based analysis. Breast Cancer Res Treat. 2018;172:133–42. <https://doi.org/10.1007/s10549-018-4877-9>.
19. Threlfall AG, Collins S, Woodman CBJ. Impact of NHS breast screening on advanced disease and mortality from breast cancer in the North West of England. Br J Cancer. 2003;89:77–80. <https://doi.org/10.1038/sj.bjc.6600842>.
20. Fracheboud J, de Koning HJ, Boer R, Groenewoud JH, Verbeek ALM, Broeders MJM, et al. Nationwide breast cancer screening programme fully implemented in the Netherlands. Breast. 2001;10:6–11. <https://doi.org/10.1054/brst.2000.0212>.
21. Paci E. Evaluation of the impact of service screening: the Florence city programme (1990–1996). Semin Breast Dis. 2007;10:64–7. <https://doi.org/10.1053/j.sembd.2007.09.002>.
22. Musolino A, Michiara M, Conti GM, Boggiani D, Zatelli M, Palleschi D, et al. Human epidermal growth factor receptor 2 status and interval breast cancer in a population-based cancer registry study. J Clin Oncol. 2012;30:2362–8. <https://doi.org/10.1200/JCO.2011.37.6434>.
23. Bulliard JL, Ducros C, Jemelin C, Arzel B, Fioretta G, Levi F. Effectiveness of organised versus opportunistic mammography screening. Ann Oncol. 2009;20:1199–202. <https://doi.org/10.1093/annonc/mdn770>.
24. Braun B, Khil L, Tio J, Krause-Bergmann B, Fuhs A, Heidinger O, et al. Differences in breast cancer characteristics by mammography screening participation or non-participation. Dtsch Arztebl Int. 2018;115:520–7. <https://doi.org/10.3238/arztebl.2018.0520>.
25. Poiseuil M, Coureau G, Payet C, Savès M, Debled M, Mathoulin-Pelissier S, et al. Deprivation and mass screening: survival of women diagnosed with breast cancer in France from 2008 to 2010. Cancer Epidemiol. 2019;60:149–55. <https://doi.org/10.1016/j.canep.2019.03.016>.
26. ResearchGate [Internet]. Studying impact of screening: large differences in stage distribution irrespective of varying definitions of advanced breast cancer. Disponible sur. [cité 14 Mai 2025], https://www.researchgate.net/publication/300034198_Studying_impact_of_screening_Large_differences_in_stage_distribution_irrespective_of_varying_definitions_of_advanced_breast_cancer.
