# Supplementary material for: Use of population-based cancer registry data to evaluate organized breast cancer screening programmes in Europe by mode of detection: a scoping review
Source: Eur J Public Health. 2026 Jun 17;36(4):ckag090. doi: 10.1093/eurpub/ckag090 (PMC13275118; doi:10.1093/eurpub/ckag090)
Supplement: ckag090_Supplementary_Data [file ckag090_supplementary_data.zip › ejph-2026-03-om-0248-File005.docx]

**Inclusion and Exclusion criteria**

| ***Inclusion Criteria For all articles*** | |
| --- | --- |
| 1 | Studies conducted within the European continent (Supplementary Material 1). |
| 2 | Studies that include a measure of cancer screening intensity (individual level, aggregated level, or proxy indicators). |
| 3 | Studies analysing Population-Based Cancer Registry (PBCR) outcomes, including incidence, disease progression at diagnosis, treatment, follow-up, or vital status. |
| 4 | Studies evaluating an established cancer screening programme defined as a public health Policy specifying at least one screening examination, a screening interval, and an eligible population. |
| ***Exclusion Criteria For all articles*** | |
| 1 | Cancers other than breast cancer |
| 2 | Simulation, methodological, theoretical, interventional papers, or papers not reporting original research |
| 3 | No link between a screening intensity measure and a PBCR outcome |
| 4 | No evaluation of the eligible population (as defined by the article) |
| ***Additional exclusion criterion - For articles set aside where no support was available*** | |
| 5 | Articles written in languages beyond the group’s language proficiency |
